# Young Adults’ Perceptions of and Intentions to Use Nicotine and Cannabis Vaporizers in Response to e-Cigarette or Vaping-Associated Lung Injury Instagram Posts: Experimental Study

**DOI:** 10.2196/46153

**Published:** 2023-09-14

**Authors:** Karla D Llanes, Pamela M Ling, Jamie Guillory, Erin A Vogel

**Affiliations:** 1 Center for Tobacco Control Research & Education University of California San Francisco San Francisco, CA United States; 2 Prime Affect Research Dublin Ireland; 3 TSET Health Promotion Research Center Stephenson Cancer Center University of Oklahoma Health Sciences Center Oklahoma City, OK United States

**Keywords:** EVALI, risk perception, nicotine, cannabis, e-cigarettes, young adult, vaping, social media, Instagram, harmful effect

## Abstract

**Background:**

Inhaling aerosolized nicotine and cannabis (colloquially called “vaping”) is prevalent among young adults. Instagram influencers often promote both nicotine and cannabis vaporizer products. However, Instagram posts discouraging the use of both products received national media attention during the 2019 outbreak of e-cigarette or vaping-associated lung injury (EVALI).

**Objective:**

This experiment tested the impact of viewing Instagram posts about EVALI, varying in image and text valence, on young adults’ perceived harmfulness of nicotine and cannabis products, perceived risk of nicotine and cannabis vaporizer use, and intentions to use nicotine and cannabis vaporizers in the future.

**Methods:**

Participants (N=1229) aged 18-25 (mean 21.40, SD 2.22) years were recruited through Qualtrics Research Services, oversampling for ever-use of nicotine or cannabis vaporizers (618/1229, 50.3%). Participants were randomly assigned to view Instagram posts from young people portraying their experiences of EVALI in a 2 (image valence: positive or negative) × 2 (text valence: positive or negative) between-subjects experiment. Positive images were attractive and aesthetically pleasing selfies. The positive text was supportive and uplifting regarding quitting the use of vaporized products. Negative images and text were graphic and fear inducing. After viewing 3 posts, participants reported the perceived harmfulness of nicotine and cannabis products, the perceived risk of nicotine and cannabis vaporizer use, and intentions to use nicotine and cannabis vaporizers in the future. Ordinal logistic regression models assessed the main effects and interactions of image and text valence on perceived harmfulness and risk. Binary logistic regression models assessed the main effects and interactions of image and text valence on intentions to use nicotine and cannabis vaporizers. Analyses were adjusted for product use history.

**Results:**

Compared to viewing positive images, viewing negative images resulted in significantly greater perceived harm of nicotine (*P*=.02 for disposable pod-based vaporizers and *P*=.04 for other e-cigarette “mods” devices) and cannabis vaporized products (*P*=.01), greater perceived risk of nicotine vaporizers (*P*<.01), and lower odds of intentions to use nicotine (*P*=.02) but not cannabis (*P*=.43) vaporizers in the future. There were no significant main effects of text valence on perceived harm, perceived risk, and intentions to use nicotine and cannabis vaporized products. No significant interaction effects of image and text valence were found.

**Conclusions:**

Negative imagery in Instagram posts about EVALI may convey the risks of vaporized product use and discourage young adults from this behavior, regardless of the valence of the post’s text. Public health messaging regarding EVALI on Instagram should emphasize the risk of cannabis vaporizer use, as young adults may otherwise believe that only nicotine vaporizer use increases their risk for EVALI.

## Introduction

The prevalence of nicotine vaporizer use (ie, e-cigarette use, commonly called nicotine “vaping”) is higher among young (versus older) adults [1] and increased between 2017 and 2020 [2]. Specifically, 6.2% of young adults aged 19 to 30 years from the national Monitoring the Future (MTF) study reported past 30-day nicotine vaporizer use in 2017 and 13.7% in 2020 [2]. Nicotine vaporizer use is appealing even to young adults who have never used a tobacco product [3] and is associated with the initiation of other tobacco products and cannabis use among young adults [4,5]. As nicotine vaporizer prevalence increased, the prevalence of inhaling aerosolized tetrahydrocannabinol (THC) (ie, “cannabis vaping”) from oil, wax, liquid, or dry cannabis leaf with portable electronic devices also increased among young adults. Among young adults aged 19 to 30 years, 6.1% and 10.8% reported past 30-day cannabis vaporizer use in 2017 and 2020, respectively [2].

Concerns about the health effects of nicotine vaporizer use rose during an outbreak of e-cigarette or Vaping Associated Lung Injury (EVALI) that made headlines in 2019 and 2020 [6] when over 2600 previously healthy, mostly younger, people in the United States developed symptoms such as coughing, chest pain, and shortness of breath [7]. Since the first case in August 2019, some required hospitalization and mechanical ventilation, and there were 68 deaths by February 2020 [7,8]. EVALI was linked to exposure to vitamin E acetate, a diluent primarily found in illicit cannabis vaporizer cartridges [6,9], although some individuals with EVALI reported using only nicotine vaporizers [6,10]. Most people who experienced EVALI reported obtaining their device or product from informal sources, such as a friend or a dealer [10].

Social media has great potential to disseminate information about diseases such as EVALI to young adults, with 84% of US young adults reporting social media use in 2021 [11]. Information about EVALI arose organically on social media, as young adults shared their experiences with the illness through posts on the popular social media app Instagram [12,13]. Instagram posts about EVALI, which have reached hundreds of thousands of Instagram users, may be captivating because Instagram allows users to share striking visual imagery alongside text [14]. Images and text may be negative (eg, graphic or fear inducing) or positive (eg, aesthetically pleasing or uplifting) in valence.

Viewing EVALI-related imagery in Instagram posts may discourage nicotine or cannabis vaporizer use, which is similar to pictorial warning labels on tobacco products. A systematic review of experimental studies concluded that compared to text-only warnings, pictorial warnings attracted greater attention, elicited negative attitudes about the product, and may reduce intentions to smoke among youth and young adults [15]. Although pictorial warnings may be more effective than text warnings, negative text may also evoke fear. Fear appeals (ie, persuasive messages that arouse fear) can result in both increased and decreased engagement in a health behavior (eg, vaporizer use). According to the Extended Parallel Process Model [16], fear appeals will encourage positive behavior change when the behavior is perceived as both harmful and avoidable. Fear-inducing imagery and text on Instagram may increase the perceived harm of vaporizer use and decrease use intentions. On the other hand, positive imagery and supportive text may bolster young adults’ confidence in their ability to avoid vaporizer use and also decrease use intentions. Experimental research is needed to understand how positively and negatively framed EVALI-related posts that have arisen on Instagram may affect young adults’ perceived harm and risk of and intentions to use nicotine and cannabis vaporizers.

We experimentally tested the impact of viewing Instagram posts about EVALI, which varied in image and text valence, on young adults’ perceived harmfulness of different vaporizer products, and perceived riskiness of and intentions to use nicotine and cannabis vaporizers. We hypothesized that (1) compared with exposure to EVALI-related Instagram posts featuring positive images (H1), exposure to Instagram posts featuring negative images would result in greater perceived risks of nicotine vaporizer use (H1a), greater perceived risks of cannabis vaporizer use (H1b), greater perceived harm of nicotine vaporizer use (H1c), greater perceived harm of cannabis vaporizer use (H1d), lower intentions to use nicotine vaporizers (H1e), and lower intentions to use cannabis vaporizers (H1f); (2) compared with exposure to EVALI-related Instagram posts featuring positive text (H2), exposure to Instagram posts featuring negative text would result in greater perceived risks of nicotine vaporizer use (H2a), greater perceived risks of cannabis vaporizer use (H2b), greater perceived harm of nicotine vaporizer use (H2c), greater perceived harm of cannabis vaporizer use (H2d), lower intentions to use nicotine vaporizers (H2e), and lower intentions to use cannabis vaporizers (H2f); (3) effects of image valence (H1) on perceived risk and harm and intentions to use nicotine vaporizers and cannabis vaporizers will be greater than effects of text valence (H2). Analyses included image valence by text valence interaction terms to explore whether the effect of image valence was dependent on text valence and vice versa.

## Methods

### Participants

We recruited participants (N=1229) aged 18 to 25 (mean 21.40, SD 2.22) years through Qualtrics Research Services, oversampling participants who reported ever using nicotine or cannabis vaporizers (618/1229, 50.3%). To be eligible, participants also had to be 18-25 years old and report using Instagram at least weekly.

### Ethics Approval

This study was reviewed and approved by the University of California, San Francisco institutional review board (IRB 11-06516). All participants read and provided electronic informed consent on Qualtrics before proceeding with the survey and random assignment to an experimental condition. Participants were assured confidentiality. The researchers received deidentified data from Qualtrics Research Services. Participants were compensated by their survey panel providers in accordance with their preexisting agreement with the panel provider.

### Design and Procedure

We randomized participants to view EVALI-related posts that varied by image valence and text valence in a 2 (image valence: positive or negative) × 2 (text valence: positive or negative) between-subjects design. Participants first completed an initial questionnaire measuring demographics, Instagram use intensity, and tobacco and cannabis vaporizer behavior (see Table 1). Next, participants viewed 3 Instagram posts concordant with their assigned experimental condition (described in “Experimental Stimuli”). After viewing all 3 Instagram posts, participants rated the perceived harmfulness of different vaporizer products, agreement with statements related to the riskiness of nicotine vaporizer and cannabis vaporizer use, and intentions to use nicotine and cannabis vaporizers.

**Table 1:** Participant Characteristics (N=1229).

Characteristics		Values
Sex assigned at birth (female), n (%)		679 (55.2)
**Gender identity, n (%)**		
	Female	618 (50.3)
	Male	543 (44.2)
	Another gender^a^	68 (5)
**Sexual identity, n (%)**		
	Straight or heterosexual	931 (78)
	Gay	32 (3)
	Lesbian	32 (3)
	Bisexual	192 (16)
	Another sexual identity^b^	42 (3)
Age (years), mean (SD)		21.40 (2.22)
**Race and ethnicity, n (%)**		
	Non-Hispanic White	666 (54.3)
	Non-Hispanic Black	161 (13.1)
	Hispanic	208 (17)
	Non-Hispanic Asian, native Hawaiian, or other Pacific Islander	119 (9.7)
	Another or unreported race or ethnicity^c^	73 (6)
**Education, n (%)**		
	Less than college degree	884 (71.93)
	College degree	331 (26.93)
	Does not wish to answer	14 (1)
**Current student status, n (%)**		
	Not currently attending school	307 (25)
	High school or general educational development classes	217 (17.7)
	Community college	225 (18.3)
	4-year college or university	401 (32.60)
	Graduate or professional school	79 (6.4)
Instagram use intensity, mean (SD)		3.51 (0.92)
**Past-month nicotine and cannabis use (yes), n (%)**		
	Cigarettes	210 (50)
	Nicotine vaporizers	237 (79.3)
	Cannabis vaporizers	301 (71.7)
**Time to first nicotine vaporizer use, n (%)**		
	Within 30 minutes of waking	122 (43.6)
	After 30 minutes	158 (56.4)
Self-perceived nicotine vaporizer addiction from 0% to 100% (%), mean (SD)		50.72 (32.07)

^a^Includes transgender female/transgender woman, transgender male/transgender man, nonbinary, gender queer, gender nonconforming, and selected “Other.”

^b^Includes selected “Other.”

^c^Includes American Indian, Alaska Native, multiple races, prefer not to answer, prefer not to answer but selected other, and prefer not to report ethnicity but selected a race.

### Experimental Stimuli

**Figure 1:**
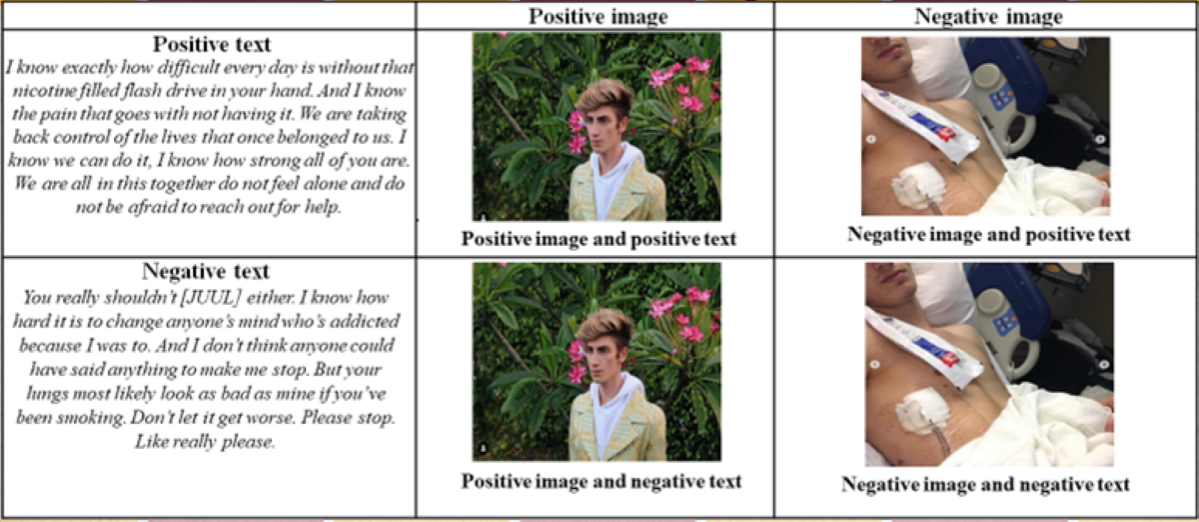
Image and text valence per condition.

We derived the 3 Instagram posts that participants viewed from images and text posted on Instagram by 3 young adults in the United States whose experiences with EVALI had been featured in news media. We contacted the young adults using Instagram direct messages to inform them of the study and gave them an opportunity to opt out of having their images and text used in research. We edited the text for brevity and clarity. Positive images were attractive and aesthetically pleasing selfies. Positive text was supportive and uplifting (Figure 1). Negative images and text were graphic and fear inducing (Figure 1). We conducted a pretest with a sample of young adults on Amazon Mechanical Turk, who rated the valence of positive and negative stimuli. See Figure 1 for an example of image valence and text valence for each of the 4 conditions, showing the 4 variations by experimental condition for 1 of the 3 social media users.

### Measures

#### Outcomes

With 4 items, participants rated the *harmfulness* [17] of 4 different products (cigarettes, disposable pod-based e-cigarettes, other nicotine vaporizer products, and marijuana or cannabis vaporizer products) following the prompt: “Imagine you use each of the products below 2 to 3 times a day, every day. How HARMFUL would this be for YOUR HEALTH?” (1=not at all harmful, 4=extremely harmful). We measured the *perceived risk of vaping* [18] with 4 items assessing agreement (1=strongly disagree, 4=strongly agree) with the following statements: (1) vaping is safer than smoking cigarettes; (2) vaping cannabis or marijuana can cause lung injuries; (3) young people are at risk of respiratory problems due to e-cigarettes and vaping; and (4) there is no hard evidence that vaping nicotine increases the risk of severe lung disease. We measured *intentions to use nicotine vaporizers* [19] by asking participants whether they thought they would vape nicotine soon (1=definitely not, 4=definitely yes). We measured *intentions to use cannabis vaporizers* by asking participants if they thought they would vape marijuana, cannabis, or THC, soon (1=definitely not, 4=definitely yes). A “definitely not” response indicated no intent; all other responses indicated potential intent [19,20].

#### Participant Characteristics

We used 1 item adapted from the Facebook Intensity Scale [21] to assess the frequency of Instagram use (never, less than once a month, monthly, a few times a month, weekly, a few times a week, daily, or several times per day). Participants also reported both ever-use (yes or no) and past-month use (0-30 days) of cigarettes, nicotine vaporizers, and cannabis vaporizers. Examples of products were provided. Past-month use was coded as any past-month use (1+ days) versus no past-month use.

Participants reported their age, sex assigned at birth, education, current student status, and race and ethnicity (coded as non-Hispanic White; non-Hispanic Black; Hispanic; Asian, including Native Hawaiian/Pacific Islander; or other/unreported race or ethnicity) (Table 1).

### Statistical Analysis

We compared participant characteristics by condition using chi-square and *F* tests and did not find significant differences, except for sexual identity (Table S1 in Multimedia Appendix 1). Ordinal logistic regression models assessed the main effects and interactions of image and text valence on the perceived risk of vaping nicotine (H1a and H2a) and cannabis (H1b and H2b) products and the perceived harm of vaping nicotine (H1c and H2c) and cannabis (H1d and H2d) products. Binary logistic regression models assessed the main effects and interactions of image and text valence on intentions to use nicotine (H1e and H2e) and cannabis (H1f and H2f) vaporizers. All models presented in Tables 2 to 5 are adjusted for ever-use of nicotine and cannabis vaporizers. We also ran sensitivity analyses for the use intentions outcomes, including only never-users of nicotine vaporizers and cannabis vaporizers, to examine whether image valence and text valence affected their susceptibility to initiating use.

## Results

### Perceived Risk of Vaporizer Use

**Figure 2:**
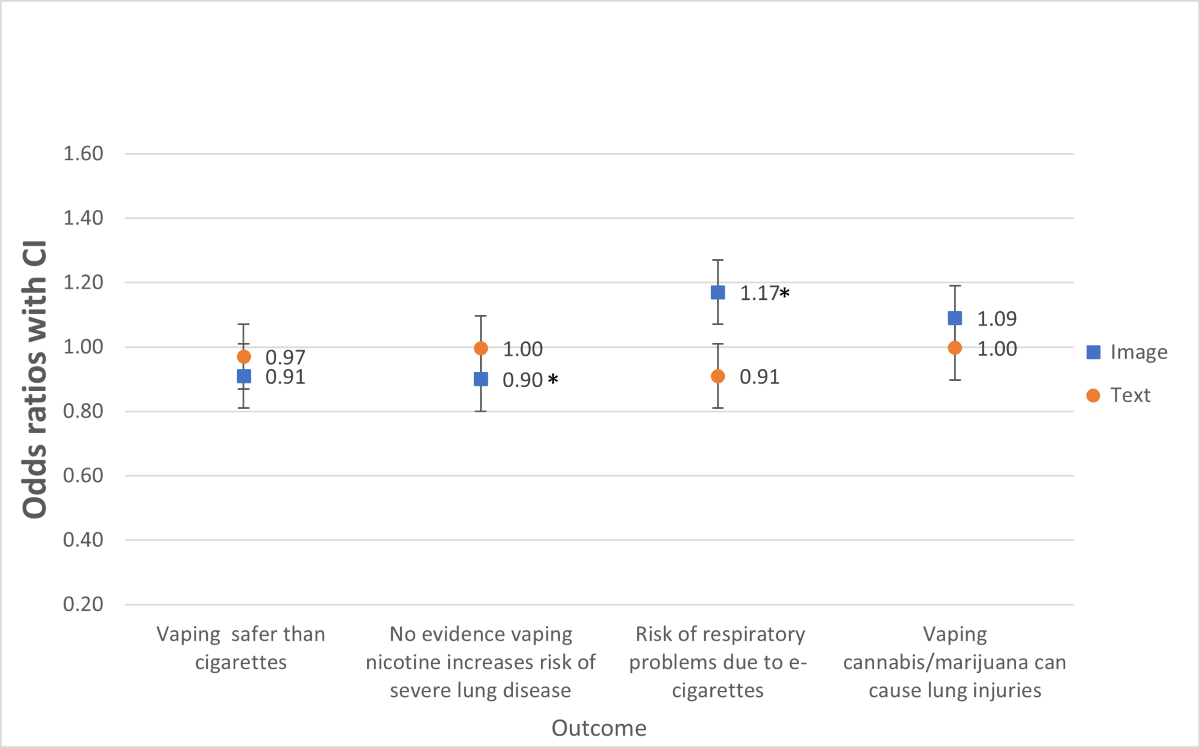
Comparing image valence (positive versus negative) and text valence on vaporizers risk perceptions and safety perceptions. The odds ratios were adjusted for ever-use of nicotine and cannabis vaporizers. *represents a significant difference at *P*<.05 for positive or negative valence (ie, image or text) comparison.

For agreement with the statement “Vaping is safer than smoking cigarettes,” there were no significant main effects of image valence. Participants who viewed negative images (odds ratio [OR] 0.90, 95% CI 0.81-0.99) were less likely to agree that “There is no hard evidence that vaping nicotine increases the risk of severe lung disease.” Participants who viewed negative, compared with positive, images (OR 1.17, 95% CI 1.05-1.30) were more likely to agree that “Young people are at risk of respiratory problems due to e-cigarettes and vaping.” For agreement with the statement “Vaping cannabis/marijuana can cause lung injuries,” there were no significant main effects of image valence. H1a was supported, but H1b was not supported. There were no significant main effects of text valence (ie, H2a and H2b were not supported) and no significant interaction effects of image and text valence for the *perceived risk of vaporizer use* statements (Table 2 and Figure 2).

**Table 2:** Ordinal logistic regression: vaporizer risk perceptions.

Predictors			Β		SE		Odds ratio (95% CI)
**Vaping is safer than smoking cigarettes^a^**							
	Negative image=+1 vs positive image=−1	−0.09		0.05		0.91 (0.82-1.01)	
	Negative text=+1 vs positive text=−1	−0.04		0.05		0.97 (0.87-1.07)	
	Image and text interaction	−0.04		0.05		0.96 (0.87-1.06)	
	*Ever vaped nicotine* (*no=0, yes=1*)^b^	−*0.74*		*0.13*		*2.09* (*1.63-2.69*)	
	Ever vaped cannabis (no=0, yes=1)	−0.21		0.13		1.23 (0.95-1.60)	
**There is no hard evidence that vaping nicotine increases the risk of severe lung disease^c^**							
	*Negative image=+1 vs positive image=* *−* *1*	−*0.11*		*0.05*		*0.90* (*0.81-0.99*)	
	Negative text=+1 vs positive text=−1	−0.004		0.05		1.00 (0.90-1.11)	
	Image and text interaction	−0.05		0.05		0.95 (0.86-1.05)	
	*Ever vaped nicotine* (*no=0, yes=1*)	−*0.67*		*0.13*		*1.95* (*1.51-2.51*)	
	Ever vaped cannabis (no=0, yes=1)	−0.06		0.14		1.06 (0.81-1.38)	
**Young people are at risk of respiratory problems due to e-cigarettes and vaping^d^**							
	*Negative image=+1 vs positive image=* *−* *1*	*0.16*		*0.05*		*1.17* (*1.05-1.30*)	
	Negative text=+1 vs positive text=−1	−0.09		0.06		0.91 (0.82-1.02)	
	Image and text interaction	−0.002		0.05		0.998 (0.897-1.11)	
	*Ever vaped nicotine* (*no=0, yes=1*)	−*0.51*		*0.14*		*0.60* (*0.459-0.79*)	
	Ever vaped cannabis (no=0, yes=1)	0.08		0.14		0.925 (0.702-1.22)	
**Vaping cannabis and marijuana can cause lung injuries^e^**							
	Negative image=+1 vs positive image=−1	0.09		0.05		1.09 (0.98-1.21)	
	Negative text=+1 vs positive text=−1	−0.003		0.05		0.997 (0.898-1.11)	
	Image and text interaction	−0.02		0.05		0.98 (0.89-1.09)	
	*Ever vaped nicotine* (*no=0, yes=1*)	−*0.38*		*0.13*		*0.69* (*0.53-0.89*)	
	*Ever vaped cannabis* (*no=0, yes=1*)	−*0.56*		*0.138*		*0.57* (*0.44-0.75*)	

^a^ Model fit: *G*^2^_5_=218.11, *P*<.05.

^b^Italic formatting represents significance at *P*<.05. Dependent Variable Question: “How much do you agree/disagree with the following statements about vaping in general?”

^c^Model fit: *G^2^*_5_=237.00, *P*<.05.

^d^Model fit: *G*^2^_5_=243.27, *P*<.05.

^e^Model fit: *G*^2^_5_=233.56, *P*<.05.

### Perceived Harmfulness

**Figure 3:**
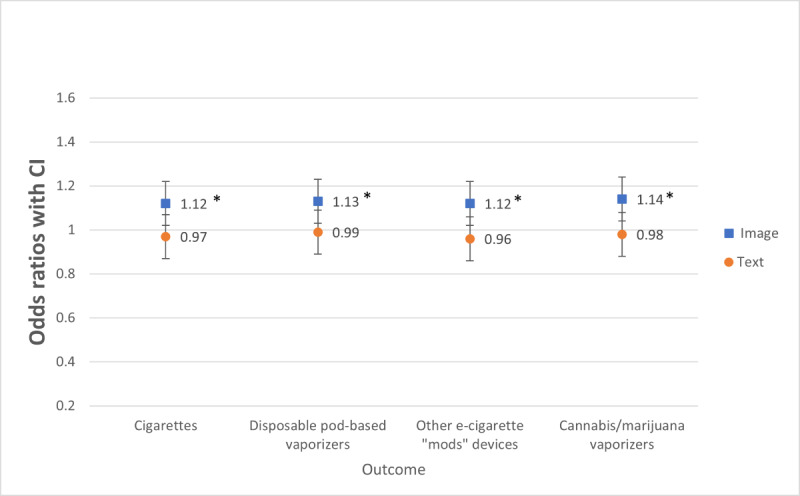
Comparing image valence (positive versus negative) and text valence on perceived harmfulness of tobacco and cannabis products. The odds ratios were adjusted for ever-use of nicotine and cannabis vaporizers. * represents a significant difference at *P*<.05 for positive or negative valence (ie, image or text) comparison.

Participants who viewed negative images, compared with positive images, rated cigarettes (OR 1.12, 95% CI 1.01-1.25), disposable pod-based nicotine vaporizers (OR 1.13, 95% CI 1.02-1.25), other e-cigarette “mod” devices (OR 1.12, 95% CI 1.01-1.24), and cannabis and marijuana vaping products (OR 1.14, 95% CI 1.03-1.26) as more harmful (*P*=.01), which support H1c and H1d. There were no significant main effects of text valence and no significant image and text valence interactions for any product harmfulness ratings (Table 3 and Figure 3); thus, H2c and H3 were not supported.

**Table 3:** Ordinal logistic regression: perceived harmfulness of product or drug.

Predictors			Β		SE	Odds ratio (95% CI)
**Cigarettes^a^**						
	*Negative image=+1 vs positive image=−1* ^b^	*0.12*		*0.05*		*1.12* (*1.01-1.25*)
	Negative text=+1 vs positive text=−1	−0.03		0.05		0.97 (0.87-1.08)
	Image and text interaction	0.07		0.05		1.08 (0.97-1.19)
	*Ever vaped nicotine* (*no=0, yes=1*)	−*0.62*		*0.13*		*0.54* (*0.42-0.70*)
	Ever vaped cannabis (no=0, yes=1)	0.07		0.14		1.08 (0.82-1.41)
**Disposable pod-based vaporizers^c^**						
	*Negative image=+1 vs positive image=−1*	*0.12*		*0.05*		*1.13* (*1.02-1.25*)
	Negative text=+1 vs positive text=−1	−0.01		0.05		0.99 (0.89-1.10)
	Image and text interaction	0.05		0.05		1.05 (0.94-1.16)
	*Ever vaped nicotine* (*no=0, yes=1*)	−*0.70*		*0.13*		*0.50* (*0.39-0.65*)
	Ever vaped cannabis (no=0, yes=1)	−0.07		0.14		0.94 (0.72-1.23)
**Other e-cigarette “mods” devices^d^**						
	*Negative image=+1 vs positive image=−1*	*0.11*		*0.05*		*1.12* (*1.01-1.24*)
	Negative text=+1 vs positive text=−1	−0.04		0.05		0.96 (0.87-1.07)
	Image and text interaction	0.02		0.05		1.02 (0.92-1.13)
	*Ever vaped nicotine* (*no=0, yes=1*)	*0.62*		*0.13*		*0.54* (*0.42-0.70*)
	Ever vaped cannabis (no=0, yes=1)	0.14		0.14		0.87 (0.67-1.14)
**Cannabis and marijuana vaporizers^e^**						
	*Negative image=+1 vs positive image=−1*	−*0.13*		*0.05*		*1.14* (*1.03-1.26*)
	Negative text=+1 vs positive text=−1	0.02		0.05		0.98 (0.89-1.09)
	Image and text interaction	0.02		0.05		1.02 (0.92-1.14)
	*Ever vaped nicotine* (*no=0, yes=1*)	−*0.63*		*0.13*		*0.53* (*0.41-0.69*)
	*Ever vaped cannabis* (*no=0, yes=1*)	−*0.75*		*0.14*		*0.47* (*0.36-0.62*)

^a^Model fit: *G*^2^_5_=203.57, *P*<.05.

^b^Italic formatting represents significance at *P*<.05. Dependent Variable Question: “Imagine you use each of the products below 2 to 3 times a day, every day. How HARMFUL would this be for YOUR HEALTH? – Product.”

^c^Model fit: *G*^2^_5_=200.24, *P*<.05.

^d^Model fit: *G*^2^_5_=208.03, *P*<.05.

^e^Model fit: *G^2^*_5_=207.89, *P*<.05.

### Nicotine and Cannabis Vaporizer Use Intentions

**Figure 4:**
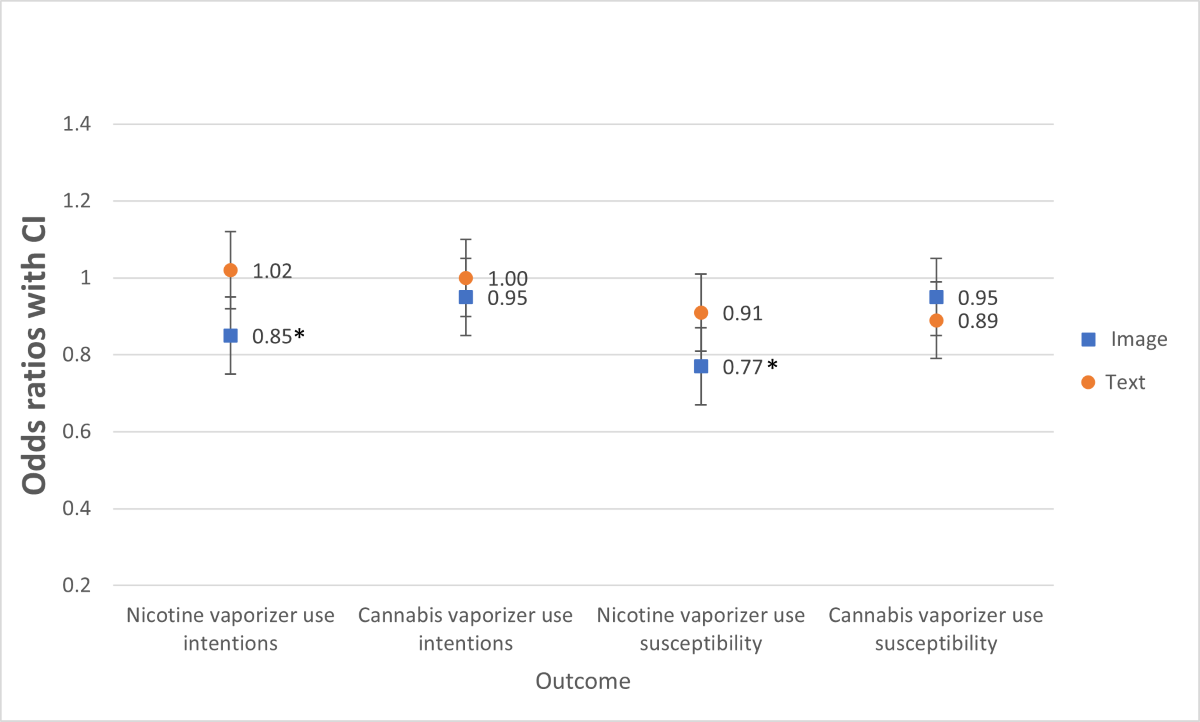
Comparing image valence (positive versus negative) and text valence on vaporizer user’s intentions to use vaporizers and nonusers susceptibility to use vaporizers. Susceptibility was measured only in a subset of participants who had never vaped. The odds ratios were adjusted for ever-use of nicotine and cannabis vaporizers. *represents a significant difference at *P*<.05 for positive or negative valence (ie, image or text) comparison.

Participants who viewed the negative, compared with positive, images (OR 0.85, 95% CI 0.74-0.97) were less likely to intend to use nicotine vaporizers (Table 4 and Figure 4), which supports H1e. There were no significant main effects of text valence and no significant image and text valence interactions for intentions to use nicotine or cannabis vaporizers, and no significant main effect of image valence on cannabis vaporizer use intentions (see Table 5 and Figure 4); therefore, H1f, H2e, and H2f were not supported.

**Table 4:** Binary logistic regression: nicotine vaporizer use intentions^a^.

Predictors	Β	SE	Odd ratio (95% CI)
*Negative image=+1 vs positive image=−1* ^b^	−*0.17*	*0.07*	*0.85* (*0.74-0.97*)
Negative text=+1 vs positive text=−1	0.02	0.07	1.02 (0.89-1.16)
Image and text interaction	−0.13	0.07	0.88 (0.77-1.01)
*Ever vaped nicotine use* (*no=0, yes=1*)	*2.00*	*0.17*	*7.36* (*5.30-10.23*)
*Ever vaped cannabis* (*no=0, yes=1*)	*0.67*	*0.16*	*1.95* (*1.43-2.66*)
Constant	−2.0	0.12	0.14 (N/A^c^)

^a^Model fit: *χ*^2^_5_=428.20, *P*<.05.

^b^Italic formatting represents significance at *P*<.05. Dependent Variable Question: “Do you think you will vape nicotine soon?”

^c^N/A: not available.

**Table 5:** Binary logistic regression: cannabis vaporizer use intentions^a^.

Predictors	Β	SE	Odd ratio (95% CI)
Negative image=+1 vs Positive image=−1	−0.06	0.07	0.95 (0.82-1.09)
Negative text=+1 vs positive text=−1	−0.01	0.07	1.00 (0.87-1.14)
Image and text interaction	−0.03	0.07	0.97 (0.85-1.12)
*Ever vaped nicotine* (*no=0, yes=1*)^b^	*1.25*	*0.16*	*3.49* (*2.57*-*4.74*)
*Ever vaped cannabis* (*no=0, yes=1*)	*1.86*	*0.17*	*6.39* (*4.62-8.85*)
Constant	−1.61	0.11	0.20 (N/A^c^)

^a^Model fit: *χ*^2^_5_=342.71, *P*<.05.

^b^Italic formatting represents significance at *P*<.05. Dependent Variable Question: “Do you think you will vape marijuana, cannabis, or THC soon?

^c^N/A: not available.

We ran sensitivity analyses including only never-users to examine their susceptibility (ie, use intentions among never-users) to initiating nicotine and cannabis vaporizer use [20]. Results were similar to those found in the full sample (see Tables 6 and 7 and Figure 4). Never-users who viewed the negative, compared with positive, images (OR 0.77, 95% CI 0.60-0.99) were less likely to be susceptible to using nicotine vaporizers. In addition, there were no significant main effects of text valence and no significant image and text valence interactions for susceptibility to nicotine or cannabis vaporizer use, and no significant main effect of image valence on cannabis vaporizer susceptibility (*P*<.05).

**Table 6:** Binary logistic regression: nicotine vaporizer use intentions (susceptibility) for never users^a^.

Predictors	Β	SE	Odd ratio (95% CI)
*Negative image=+1 vs positive image=* *−* *1* ^b^	−*0.26*	*0.13*	*0.77* (*0.60-0.99*)
Negative text=+1 vs. positive text=−1	−0.10	0.13	0.91 (0.71-1.17)
Image and text interaction	−0.13	0.13	0.88 (0.69-1.13)
Constant	−1.99	0.13	0.14 (N/A^c^)

^a^Model fit: *χ*^2^_3_=5.45, *P*=.14.

^b^Italic formatting represents significance at *P*<.05. Dependent Variable Question: “Do you think you will vape nicotine soon?” Never users were classified as individuals who never tried vaping (both nicotine and cannabis), even just 1 time, in their entire lives.

^c^N/A: not available.

**Table 7:** Binary logistic regression: cannabis vaporizer use intentions (susceptibility) for never users^a^.

Predictors	Β	SE	Odd ratio (95% CI)
Negative image=+1 vs positive image=−1	−0.05	0.11	0.95 (0.77-1.18)
Negative text=+1 vs positive text=−1	−0.17	0.11	0.89 (0.72-1.10)
Image and text interaction	−0.06	0.11	0.95 (0.77-1.17)
Constant	−1.58	0.11	0.21 (N/A^b^)

^a^Model fit: χ^2^_3_=1.59, *P*=.66.

^b^N/A: not available. Dependent Variable Question: “Do you think you will vape marijuana, cannabis, or THC soon?"

## Discussion

### Principal Findings

In this study, we found that EVALI-related Instagram posts with negative imagery increased young adults’ perceived harmfulness of nicotine and cannabis vaporizer products, increased perceived risks of nicotine vaporizer products, and decreased intentions to use nicotine vaporizers. Regardless of the Instagram post text, posts about EVALI that use negative imagery may better convey the harms of nicotine vaporizer use, as well as discourage young adults from nicotine vaporizer use, compared with those using positive imagery. These findings suggest that in Instagram posts, negative imagery may be more powerful than positive imagery and imagery may have a stronger impact than text on perceived risk of, perceived harmfulness of, and intentions to use nicotine vaporizers. While we are not aware of other studies comparing the valence of image and text in tobacco-related messages on Instagram, these findings are consistent with experimental studies on graphic warning labels, which found images were more effective than text in increasing perceived harmfulness of nicotine and cannabis vaporizer use [22]. In addition, other research has found that images are more engaging and that viewers pay more attention to the images than text on Instagram [23].

In this study, text was less effective in influencing general harm perceptions, perceived risk of vaporizer use, and use intentions than images (supporting H3). This finding is somewhat inconsistent with the literature finding that text-based fear appeal messages are effective [16]. Using a social media platform where the content is mostly images may have influenced the findings. Study participants may have paid more attention to the images rather than the text, resulting in a null effect of text valence. In addition, in this study, the text was derived from real Instagram posts to increase ecological validity. The text specifically written to induce fear may have had a stronger effect on perceived harms. However, a pretest of experimental stimuli confirmed that young adults did perceive the negative valence text as scary. The findings of this study have implications for how social media posts with images and text can impact risk perceptions and intentions to use nicotine and cannabis vaporizers. Campaigns using highly visual media like Instagram could use primarily negative images, which were more effective than positive images at conveying health harms. Imagery may need to explicitly portray the risks of cannabis vaporizers in addition to the risks of nicotine vaporizer use.

The study also found that negative imagery was associated with increased general perceived harmfulness of cannabis vaporizer products, although participants who saw negative images were not more likely to agree that vaping cannabis causes lung injuries. In addition, those who viewed negative images reported lower nicotine vaporizer use intentions but not lower cannabis vaporizer use intentions. The lack of effect on cannabis perceptions may be because the link between EVALI and illicit cannabis cartridges was found after the initial reports of the outbreak, which at the time communicated exposure to an unknown substance [7]. The social media users in this study referred to nicotine products in their Instagram posts, which may have increased the posts’ impact on intentions to use nicotine vaporizers. In addition, respondents may not have connected the disease to cannabis vaporizers. The finding that negative images affected nicotine intentions, but not cannabis intentions, might also be because young adults view nicotine and cannabis differently. Cannabis has a strong association with medicine and therapeutics [24,25]; therefore, different messaging or additional education interventions may be needed to increase knowledge about the specific risks of cannabis vaporizer use.

### Limitations and Future Directions

This study has several limitations. First, we recruited a web-based convenience sample, which may not be representative of all US young adults. Yet, quota sampling increased the sample diversity to match US census data. Second, we used real Instagram posts about EVALI from young adults with lived experience. Although this approach maximized ecological validity, it resulted in some loss of experimental control over the images and text.

The study results suggest that negative images from young adult social media users’ posts may be an effective way to communicate the harms of nicotine vaporizer use. Youth-oriented campaigns like “Truth” and Food and Drug Administration’s (FDA) “The Real Cost” have worked with social media influencers to encourage tobacco cessation in social media [26,27]. Negative imagery on an Instagram account from an authority figure, such as the Centers for Disease Control and Prevention, or from an account that frequently uses negative imagery, such as the FDA’s “The Real Cost” campaign [26], might be perceived differently. Future research could address whether the context of messages in different types of accounts or posted by different types of influencers (eg, those known for promoting health compared to those known for other reasons) will reduce smoking and vaporizer use. Comparing the posts from individual social media users to those posted by an organization or health authority on social media may reveal differential effects due to the source of the content.

Third, a more realistic Instagram interface, such as allowing users to scroll Instagram posts on a mobile phone, could more closely match how the posts would be viewed if individuals were using Instagram. To further increase ecological validity, future studies could post experimental stimuli on an Instagram account for participants to view. Also, the placement of the text below the images is commonly used on Instagram posts and may impact attention to images. Future studies might examine the impact of simple text-only messages placed in the image field of Instagram posts to increase attention to both images and text.

Lastly, our study was cross-sectional and did not explore the long-term impact of images and text messages on perceptions or actual vaping behavior. A longitudinal design would allow us to assess if the messages reduced nicotine and cannabis vaporizer use, unintentionally increased nicotine and cannabis vaporizer use, or both. As the use of cannabis and nicotine continues to be common, these messages need to address the use of nicotine and cannabis vaporizer products. More research is needed to understand why cannabis vaporizers were perceived as less risky than nicotine vaporizers and whether the perceived riskiness of vaporizers changes over time after exposure to antivaporizer messages. Although cannabis legalization is now widespread across the United States [28], young adults may have had more exposure to antinicotine vaporizer messaging. In 2021, 75% of US middle and high school students reported exposure to an antitobacco campaign in the past year [29]. Public health campaigns for youth and young adults should also attend to the risks of cannabis vaporizer use.

### Conclusions

Instagram posts that use negative imagery may discourage young adults from nicotine vaporizer use. Negative imagery in public education campaigns and on vaporizer product warning labels may better convey harms than text. Young adult Instagram users may be promising partners for communicating messages about health risks or the harms of consumer products. In 2016, the FDA adopted a policy requiring the advertisements of nicotine vaporizers in media (eg, social media) to include a warning statement about the addictiveness of nicotine [30,31]. The FDA could implement graphic warning labels for social media posts advertising cigarettes and consider them for nicotine vaporizers to discourage use. Negative images from Instagram posts in this study affected nicotine but not cannabis risk perceptions. Different messages may be needed to convey the risks of cannabis vaporizer use, as cannabis is perceived as medicinal, and legalization may increase perceptions of cannabis safety. Messages conveying the hazards of vitamin E acetate in illicit cannabis vaporizer cartridges may be needed to discourage cannabis vaporizer use. Since some EVALI cases were linked solely to nicotine vaping, care should be taken not to portray any form of vaporizer product use as risk free.

## Appendix

Multimedia Appendix 1 Participant characteristics by experimental condition.

## Abbreviations

EVALI: e-cigarette or vaping-associated lung injury
FDA: Food and Drug Administration
MTF: Monitoring the Future
OR: odds ratio
THC: tetrahydrocannabinol

## Data Availability

The data sets generated and analyzed during this study are available from the corresponding author on reasonable request.
