# Supplementary material for: Young Adults’ Perceptions of and Intentions to Use Nicotine and Cannabis Vaporizers in Response to e-Cigarette or Vaping-Associated Lung Injury Instagram Posts: Experimental Study
Source: J Med Internet Res. 2023 Sep 14;25:e46153. doi: 10.2196/46153 (PMC10540020; doi:10.2196/46153)
Supplement: Multimedia Appendix 1 [file jmir_v25i1e46153_app1.docx]

|  | Group 1  (*n*=310) | Group 2 (*n*=304) | Group 3  (*n*=304) | Group 4 (*n*=311) | Overall  (*N*=1229) | *p*-value |
| --- | --- | --- | --- | --- | --- | --- |
| Sex assigned at birth (n/% female) | 180  (58.1%) | 168  (55.3%) | 159  (52.3%) | 172 (55.3%) | 679  (55.2%) | .560 |
| Gender identity |  |  |  |  |  | .817 |
| Female | 166  (53.5%) | 153  (50.3%) | 144  (47.4%) | 155 (49.8%) | 618  (50.3%) |  |
| Male | 129  (41.6%) | 133 (43.8%) | 144 (47.4%) | 137 (44.1%) | 543  (44.2%) |  |
| Another gender^a^ | 15  (4.8%) | 18  (5.9%) | 16  (5.3%) | 19  (6.1%) | 68  (5.5%) |  |
| Sexual identity |  |  |  |  |  | .047* |
| Straight/heterosexual | 223  (71.9%) | 234  (77%) | 243 (79.9%) | 231  (74.3%) | 931  (78.0%) |  |
| Gay | 5  (1.6%) | 12  (3.6%) | 10  (3.3%) | 5  (1.6%) | 32  (2.6%) |  |
| Lesbian | 10  (3.2%) | 11  (3.6%) | 5  (1.6%) | 6  (1.9%) | 32  (2.6%) |  |
| Bisexual | 57  (18.4%) | 41  (13.5%) | 40  (13.2%) | 54  (17.4%) | 192  (15.6%) |  |
| Another sexual  identity^b^ | 15  (4.8%) | 6  (2%) | 6  (2%) | 15  (4.8%) | 42  (3.4%) |  |
| Age (M/SD) | 21.27  (2.21) | 21.63 (2.17) | 21.32  (2.17) | 21.37  (2.29) | 21.40  (2.22) | .199 |
| Race and ethnicity |  |  |  |  |  | .443 |
| Non-Hispanic White | 171  (55.2%) | 157  (51.8%) | 172  (56.6%) | 166  (53.5%) | 666  (54.3%) |  |
| Non-Hispanic Black | 43  (13.9) | 46  (15.2%) | 33  (10.9%) | 39  (12.6%) | 161  (13.1%) |  |
| Hispanic | 52  (16.8%) | 47  (15.5%) | 55  (18.1%) | 54  (17.4%) | 208  (17%) |  |
| Non-Hispanic Asian,  Native Hawaiian, or  Other Pacific Islander | 30  (9.7%) | 28  (9.2%) | 33  (10.9%) | 28  (9%) | 119  (9.7%) |  |
| Another or unreported race or ethnicity^c^ | 14  (4.5%) | 25  (8.3%) | 11  (3.6%) | 23  (7.4%) | 73  (5.9%) |  |
| Education |  |  |  |  |  | .685 |
| Less than college  degree | 228 (73.55%) | 215 (70.72%) | 217 (71.38%) | 224 (72.03%) | 884 (71.93%) |  |
| College degree | 80 (25.81%) | 86 (28.29%) | 81  (26.64%) | 84 (27.01%) | 331 (26.93%) |  |
| Does not wish to  answer | 2  (0.65%) | 3  (0.99%) | 6  (1.97%) | 3  (0.96%) | 14  (1.14%) |  |
| Current student status |  |  |  |  |  | .539 |
| Not currently  attending  school | 80  (25.8%) | 78  (25.7%) | 77  (25.3%) | 72  (23.2%) | 307  (25%) |  |
| High school or GED  classes | 47  (15.2%) | 57  (18.8%) | 49  (16.1%) | 64  (20.6%) | 217  (17.7%) |  |
| Community college | 65  (21%) | 48  (15.8%) | 54  (17.8%) | 58  (18.6%) | 225  (18.3%) |  |
| 4-year college or  university | 101 (32.60%) | 101  (33.2%) | 108  (35.5%) | 91  (29.3%) | 401 (32.60%) |  |
| Graduate or  professional school | 17  (5.5%) | 20  (6.6%) | 16  (5.3%) | 26  (8.4%) | 79  (6.4%) |  |
| Instagram use intensity (M/SD) | 3.54  (.90) | 3.47  (.90) | 3.50  (.94) | 3.55  (.93) | 3.51  (.92) | .653 |
| Past-month nicotine and cannabis use (n/% yes) |  |  |  |  |  |  |
| Cigarettes | 52  (48.1%) | 53  (57.6%) | 49  (46.7% | 56  (48.7%) | 210  (50%) | .420 |
| Nicotine vaporizers | 59  (72.0%) | 56  (86.2%) | 60  (85.7%) | 62  (75.6%) | 237  (79.3%) | .072 |
| Cannabis vaporizers | 88  (71.5%) | 68  72.3%) | 69  (74.2%) | 76  (69.1%) | 301  (71.7%) | .879 |
| Time to first nicotine vaporizer use |  |  |  |  |  | .239 |
| Within 30 minutes of  waking | 28  (42.4%) | 24  (34.3%) | 35  (46.7%) | 35  (50.7%) | 122  (43.6%) |  |
| After 30 minutes | 38  (57.6%) | 46  (65.7%) | 40  (53.3%) | 34  (49.3%) | 158  (56.4%) |  |
| Self-perceived nicotine vaporizer addiction from 0-100% (M/SD) | 53.54% (33.57%) | 45.2% (31.17%) | 53.28% (30.37%) | 50.90% (33.28%) | 50.72% (32.07%) | .386 |

*Note:* Percentages are of complete cases. Positive image and positive text= Group 1, positive image and negative text= Group 2, negative image and negative text= Group 3, and negative image and positive text=Group 4. We tested for differences in participant characteristics by condition using χ² and *F*-tests. Significance differences between conditions at the *p*<.05 are indicated by an *.

^a^ Includes Trans female/Trans woman, Trans male/ Trans man, Non-binary, Gender queer, Gender non-conforming, and selected “Other”

^b^ Includes selected “Other”

^c^ Includes American Indian, Alaska Native, multiple race(s), prefer not to answer, prefer not to answer but selected other, and prefer not to report ethnicity but selected a race.
